# Supplementary material for: R-propranolol is a small molecule inhibitor of the SOX18 transcription factor in a rare vascular syndrome and hemangioma
Source: eLife. 2019 Jul 30;8:e43026. doi: 10.7554/eLife.43026 (PMC6667216; doi:10.7554/eLife.43026)
Supplement: Figure 3—source data 1. [file elife-43026-fig3-data1.docx]

**Table1**

| **Supplemental Table for Figure 3C (*p*-values)** | | | | | | |
| --- | --- | --- | --- | --- | --- | --- |
| **Control versus VEGF-B** | | | | | | |
| **CD31** | **CDH5** | **NOTCH1** | | **VEGFR1** | | **PLXND1** |
| 0.0017 | .0026 | 0.0001 | | 0.00002 | | 0.00002 |
|  | | | | | | |
| **VEGF-B versus VEGF-B + aspirin** | | | | | | |
| **CD31** | **CDH5** | **NOTCH1** | **VEGFR1** | | **PLXND1** | |
| 0.1808 | 0.0546 | 0.0472 | 0.0537 | | 0.0170 | |
|  | | | | | | |
| **VEGF-B versus VEGF-B +Sm-4** | | | | | | |
| **CD31** | **CDH5** | **NOTCH1** | **VEGFR1** | | **PLXND1** | |
| 0.0061 | 0.0002 | 0.0001 | 0.0006 | | 0.00002 | |
|  | | | | | | |
| **VEGF-B versus VEGF-B + propranolol** | | | | | | |
| **CD31** | **CDH5** | **NOTCH1** | **VEGFR1** | | **PLXND1** | |
| 0.0051 | 0.0020 | 0.0000005 | 0.0007 | | 0.00002 | |
|  | | | | | | |
| **VEGF-B versus VEGF-B + R+ enantiomer** | | | | | | |
| **CD31** | **CDH5** | **NOTCH1** | **VEGFR1** | | **PLXND1** | |
| 0.0075 | 0.0025 | 0.000002 | 0.0006 | | 0.00004 | |
|  | | | | | | |
| **VEGF-B versus VEGFB + S- enantiomer** | | | | | | |
| **CD31** | **CDH5** | **NOTCH1** | **VEGFR1** | | **PLXND1** | |
| 0.0059 | 0.0002 | 0.0041 | 0.0027 | | 0.0003 | |
|  | | | | | | |
| **VEGF-B + propranolol versus VEGF-B + R+ enantiomer** | | | | | | |
| **CD31** | **CDH5** | **NOTCH1** | **VEGFR1** | | **PLXND1** | |
| 0.0061 | 0.2558 | 0.3289 | 0.0111 | | 0.3287 | |
|  | | | | | | |
| **VEGF-B + propranolol versus VEGF-B + S- enantiomer** | | | | | | |
| **CD31** | **CDH5** | **NOTCH1** | **VEGFR1** | | **PLXND1** | |
| 0.0174 | 0.0082 | 0.0037 | 0.0005 | | 0.0043 | |
|  | | | | | | |
| **VEGF-B R+ enantiomer versus VEGFB_ S-enantiomer** | | | | | | |
| **CD31** | **CDH5** | **NOTCH1** | **VEGFR1** | | **PLXND1** | |
| 0.0522 | 0.0280 | 0.0007 | 0.00004 | | 0.0133 | |
|  | | | | | | |
| **VEGF-B versus VEGF-B +Sm-4** | | | | | | |
| **CD31** | **CDH5** | **NOTCH1** | **VEGFR1** | | **PLXND1** | |
| 0.0061 | 0.0002 | 0.0001 | 0.0006 | | 0.00002 | |
|  | | | | | | |
| **VEGF-B versus VEGF-B + aspirin** | | | | | | |
| **CD31** | **CDH5** | **NOTCH1** | **VEGFR1** | | **PLXND1** | |
| 0.1808 | 0.0546 | 0.0472 | 0.0537 | | 0.0170 | |
