# Supplementary material for: R-propranolol is a small molecule inhibitor of the SOX18 transcription factor in a rare vascular syndrome and hemangioma
Source: eLife. 2019 Jul 30;8:e43026. doi: 10.7554/eLife.43026 (PMC6667216; doi:10.7554/eLife.43026)
Supplement: Transparent reporting form [file elife-43026-transrepform.pdf]

eLIFE
1st Floor
24 Hills Road
Cambridge CB2 1JP, UK
P 01223 855340
W elifesciences.org
T @elife

eLife's transparent reporting form

We encourage authors to provide detailed information *within their submission* to facilitate the interpretation and replication of experiments. Authors can upload supporting documentation to indicate the use of appropriate reporting guidelines for health-related research (see EQUATOR Network), life science research (see the BioSharing Information Resource), or the ARRIVE guidelines for reporting work involving animal research. Where applicable, authors should refer to any relevant reporting standards documents in this form.

If you have any questions, please consult our Journal Policies and/or contact us: editorial@elifesciences.org.

Sample-size estimation

- You should state whether an appropriate sample size was computed when the study was being designed
- You should state the statistical method of sample size computation and any required assumptions
- If no explicit power analysis was used, you should describe how you decided what sample (replicate) size (number) to use

Please outline where this information can be found within the submission (e.g., sections or figure legends), or explain why this information doesn't apply to your submission:

Re-human data in the manuscript this does not apply since we report 1 clinical case for an ultra-rare syndrome only a dozen of patients worldwide.
For experiments in Figure 2 and 3, we did not perform an explicit power analysis. We relied on past experiments using hemangioma- derived stem cells and mouse pre-clinical work where 3-5 biological replicates and 5-10 animals per group respectively were sufficient to detect differences. This is not explicitly stated in the manuscript text.

Replicates

- You should report how often each experiment was performed
- You should include a definition of biological versus technical replication
- The data obtained should be provided and sufficient information should be provided to indicate the number of independent biological and/or technical replicates
- If you encountered any outliers, you should describe how these were handled
- Criteria for exclusion/inclusion of data should be clearly stated
- High-throughput sequence data should be uploaded before submission, with a private link for reviewers provided (these are available from both GEO and ArrayExpress)

Please outline where this information can be found within the submission (e.g., sections or figure legends), or explain why this information doesn't apply to your submission:

Figure 1C and D and figure 2B (to assess the effect of beta-blockers on ECFC survival and luciferase assay), there were 3 independent biological replicates performed for each experiment. For figure 1E and figure supplemental figure 1 there were at least 4 biological replicates (i.e. totally independent experiments in terms of cell seeding, transfection and drug treatment) performed for each experiment. It is stated in the figure legend that no clear outliers were encountered.

Figure 2A, figure 2 supplemental 2 and figure 2 supplemental 2A and 2B, data obtained for the mouse were analysed using a number of animal per group ranging from n=3-10, of note each animals has a pair of eyes which 2 technical replicates per animal (n=3 -> 6 corneas; n=10-> 20 corneas). These numbers are indicated on the figures themselves.

For figure 2C and figure 2 supplemental figure 2C analysis of the protein pair by ALPHAScreen assay was performed in 3 different biological experiment with 3 technical replicates. This is described in the method section and the figure legend page 20.

Figure 3B represents data from 8 biological replicates. The biological replicates were performed with hemangioma stem cells isolated from 4 different proliferating phase hemangioma specimens removed between ages 3 and 9 months of age. This is stated in the results section on page 8 and page 20 in the figure legend. To verify technical replication, each PCR reaction was performed in triplicate. Values were averaged and used as one biological replicate.

Figure 3C represents data from 4 biological replicates using hemangioma stem cells isolated from two different proliferating phase hemangiomas removed at 3 and 6 months. This is stated in the results section on page 20 in the figure legends. To verify technical replication, each PCR reaction was performed in triplicate. Values were averaged and used as one biological replicate.

Figure 3 Supplemental Figure 1 shows one biological replicate where SOX18 mRNA levels were measured in four different cell populations isolated from a single hemangioma tumor. This was repeated on the same cell populations isolated from a second hemangioma, which confirmed SOX18 expression in hemangioma stem cells and endothelial cells.

Figure 3 Supplemental Figure 2 represents data from 8 biological replicates using hemangioma stem cells isolated from the same hemangioma tumors as in Figure 3B.

eLife Sciences Publications, Ltd is a limited liability non-profit non-stock corporation incorporated in the State of Delaware, USA, with company number 5030732, and is registered in the UK with company number FC030576 and branch number BR015634 at the address 1st Floor, 24 Hills Road, Cambridge CB2 1JP | August 2014

Statistical reporting

- Statistical analysis methods should be described and justified
- Raw data should be presented in figures whenever informative to do so (typically when N per group is less than 10)
- For each experiment, you should identify the statistical tests used, exact values of N, definitions of center, methods of multiple test correction, and dispersion and precision measures (e.g., mean, median, SD, SEM, confidence intervals; and, for the major substantive results, a measure of effect size (e.g., Pearson's r, Cohen's d)
- Report exact p-values wherever possible alongside the summary statistics and 95% confidence intervals. These should be reported for all key questions and not only when the p-value is less than 0.05.

Please outline where this information can be found within the submission (e.g., sections or figure legends), or explain why this information doesn't apply to your submission:

For Figure 1D and 1E, and figure 1 supplemental figure 1A,B and D (qRT-PCR analysis of ADRB KD), the statistical analysis was done using unpaired two-tailed t test.

In Figure 1C and figure 1 supplemental figure 1C (ECFC with beta-blockers experiment) data presented as SD and Mann-Whitney non-parametric T-Test used to determine statistical significance.

Figure 2A, figure 2 supplemental 2 and figure 2 supplemental 2A and 2B (in vivo mouse work), data obtained for the mouse were analysed using a 2 way ANOVA Tukey multiple comparisons. Data shown is mean ± SEM, n-number is indicated in graph or image and figure legends.

Figure 2B (luciferase assay) statistical analysis was performed using a one-way ANOVA with Bonferroni post-hoc test.

For figure 2C and figure 2 supplemental figure 2C statistical analysis of multiple protein pair by ALPHAScreen assay was performed using 2 way ANOVA Sidak's multiple comparison test.

Data in Figures 3B, 3C and Figure 3 Supplemental Figure 1 and 2 - performed with hemangioma stem cells isolated from different patient hemangiomas - were standardized as described Willems et al. Analytical Biochemistry379 (2008) 127-129. This statistical method applies log transformation, mean centering and auto-scaling to high variability biological replicates due to differences in cellular source (i.e. different patients). Data were analyzed by one-way ANOVA, Fisher Tests, and two-tailed two independent sample T-Tests. Statistical programs were from Excel and XLStat Pro.

Figure 3B can be changed to individual raw data points since N=8
We formatted Figure 3 Supplemental with individual raw data points but Figure 2 it was very cluttered looking because it entails 160 data points. We think it looks better as a bar graph but can change the format to be consistent.

(For large datasets, or papers with a very large number of statistical tests, you may upload a single table file with tests, Ns, etc., with reference to sections in the manuscript.)

Group allocation

- Indicate how samples were allocated into experimental groups (in the case of clinical studies, please specify allocation to treatment method); if randomization was used, please also state if restricted randomization was applied
- Indicate if masking was used during group allocation, data collection and/or data analysis

Please outline where this information can be found within the submission (e.g., sections or figure legends), or explain why this information doesn't apply to your submission:

Here we have 1 clinical case for an ultra-rare disorder hence there is no group allocation.

Additional data files ("source data")

- We encourage you to upload relevant additional data files, such as numerical data that are represented as a graph in a figure, or as a summary table
- Where provided, these should be in the most useful format, and they can be uploaded as "Source data" files linked to a main figure or table
- Include model definition files including the full list of parameters used
- Include code used for data analysis (e.g., R, MatLab)
- Avoid stating that data files are "available upon request"

Please indicate the figures or tables for which source data files have been provided:

Most of our data appear as images with fluorescent signals or box plot – we can provide the raw data if the manuscript is accepted.
